# Primary right atrium angiosarcoma mimicking pericarditis

**DOI:** 10.1186/1477-7819-5-120

**Published:** 2007-10-22

**Authors:** Marina Kontogiorgi, Demetrios Exarchos, Christos Charitos, Ioannis Floros, Demetra Rontogianni, Charis Roussos, Christina Routsi

**Affiliations:** 1 Department of Critical Care, Medical School of Athens University, Evangelismos Hospital, Athens Greece; 2 Department of Radiology, Evangelismos Hospital, Athens, Greece; 3 Department of Cardiothoracic Surgery, Evangelismos Hospital, Athens, Greece; 4 Department of Pathology, Evangelismos Hospital, Athens, Greece

## Abstract

**Background:**

Primary cardiac neoplasms occur rarely and most of them are benign. Malignant tumors including angiosarcoma are extremely rare and have a non specific clinical presentation and a poor prognosis.

**Case presentation:**

We present a case of a young male who was transferred to our hospital because of shock and multiple organ failure after a complicated pericardial biopsy. During the previous seven months he presented with recurrent episodes of pericardial effusions and tamponade. Chest computed tomography revealed a mass in the right atrium, infiltrating the myocardium and pericardium. During emergency surgery that followed, the patient died because of uncontrolled hemorrhage. Autopsy revealed the mass of the right atrium, which was identified on histological examination as primary cardiac angiosarcoma.

**Conclusion:**

This case highlights the difficulties both in early diagnosis and in the management of patients with cardiac angiosarcoma.

## Background

Primary tumors of the heart are extremely rare and the majority of them are benign [1-7]. Angiosarcoma is the most common primary malignant tumor in adults. It is a highly aggressive tumor characterized by a predilection in the right side of the heart, a short clinical course and a fatal outcome. Because of nonspecific clinical presentation early diagnosis is difficult. We report a case of right atrial angiosarcoma that presented with recurrent pericardial effusions and cardiac tamponade.

## Case presentation

A 29-year-old male patient was transferred to our Intensive Care Unit (ICU) from another hospital, because of shock and multiple organ failure after a complicated pericardial biopsy. Seven months before, after a syncopal episode, he had been diagnosed with cardiac tamponade diagnosed by a transthoracic echocardiogram (TEE). Subxiphoidal drainage of 700 ml hemorrhagic fluid was performed resulting in hemodynamic stabilization. Cytologic examination of the fluid was negative for malignancy. Pericarditis was diagnosed caused by chlamydia; azithromycin and anti-inflammatory drugs were administered. For the next four months the patient was relatively well except for complain of easy fatigue. From that point on, he gradually presented dyspnea on exertion, along with pain in the back and spinal column. He also had two syncopal episodes. A new TTE showed recurrence of the pericardial effusion. Chest computed tomography (CT) scan disclosed a confluent mass of the heart with myocardial and pericardial infiltration. Thoracotomy and biopsy was then decided. Trauma of the cardiac cavity occurred intraoperatively, followed by hypovolemic shock and two episodes of cardiac arrest. Subsequently, he was transferred to our tertiary hospital for further cardiosurgical treatment.

**Figure 1:**
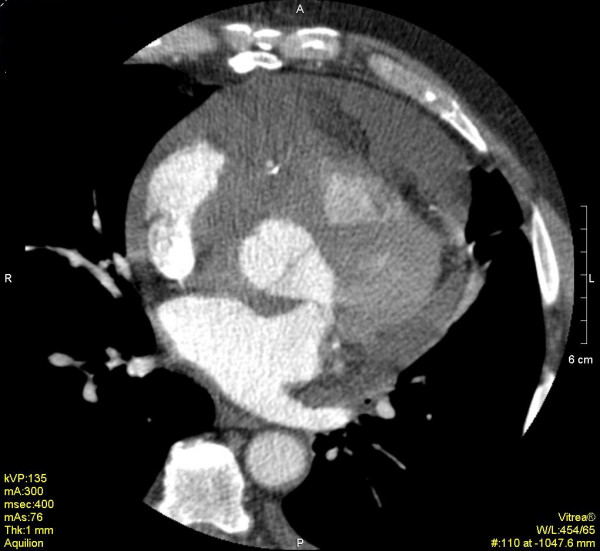
Multi-slice 16 row ECG- gated cardiac CT reveals an irregular mass in the right atrium and a moderate pericardial effusion.

On admission, the patient was mechanically ventilated, hemodynamically unstable requiring vasoactive drugs, with a temperature of 39°C. Main abnormal laboratory findings were: serum glutamic oxaloacetic transaminase (SGOT) 555 U/l, glutamic pyruvic transaminase (SGPT) 800 U/l, bilirubin 25 mg/dl, creatinine 7 mg/dl, urea 330 mg/dl, aPTT 140 sec, and INR 3.4. A continuous veno-venous hemofiltration was immediately started. Right catheterization revealed equalization of diastolic pressures. Chest CT scan revealed an irregular heterogenous mass of the right atrium with ill defined margins, infiltrating the myocardium and pericardium on the outer wall of the atrium, the inter-atricular septum, and also a pericardial effusion (Figure 1). An urgent operation was then performed. Unfortunately, the patient died during surgery due to uncontrolled hemorrhage.

**Figure 2:**
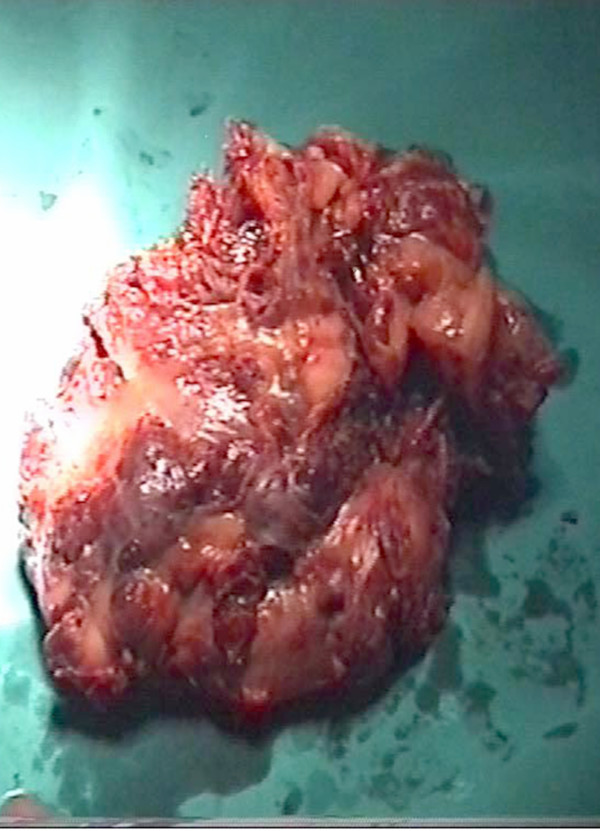
The infiltrating lesion of the pericardium-pericardium by a reddish brown neoplastic tissue.

**Figure 3:**
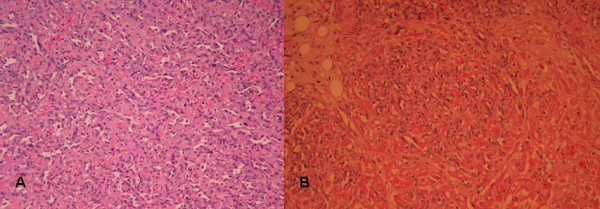
3a Infiltration of the cardiac muscle by angiosarcoma (H+E × 100) and 3b neoplastic channels lined by neoplastic endothelium (H+E × 200).

**Figure 4:**
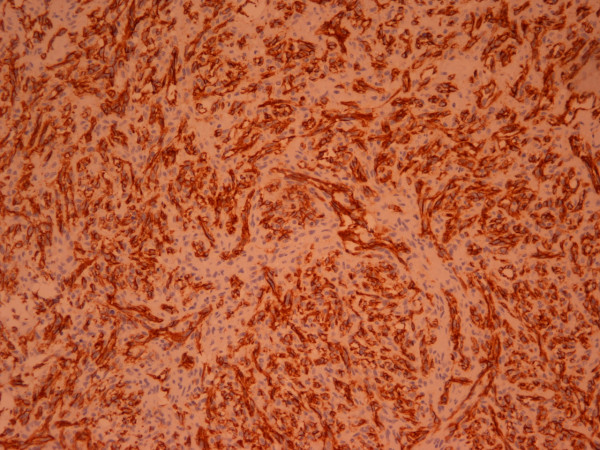
The neoplastic cells express the endothelial marker CD31 (En Vision × 150).

At autopsy, an irregular shaped, reddish brown tissue was found, 13 × 12 × 4 cm in size, covering the exterior surface of the heart (Figure 2). On opening the cardiac cavities a superficial spreading of the neoplasm to the right atrium was noted. Right ventricle, left cavities and valves presented no alterations. Microscopically, the tumor showed immunomorphological characteristics of a well-differentiated angiosarcoma (Figure 3). Neoplastic cells expressed endothelial markers positive for CD31, CD34 antibodies, and factor VII (Figure 4). Proliferation index Ki-67 assessed was 15%. Testing for HHV-8 virus was negative.

## Discussion

Primary tumors of the heart are rare with an incidence ranging from 0.0017% to 0.0033% in reported autopsy series and the majority of them are benign [1]. Angiosarcoma is the most common primary cardiac malignant tumor and is extremely rare. It is seen more commonly to males than females, usually between the third and fifth decade of life [1-5].

The location of cardiac tumors varies by the type of tumor [6]. Malignant tumors are located mainly in the right side of the heart; ninety percent of angiosarcomas are located in the right atrium and there is a high proportion of pericardial involvement [5,6]. This predilection for the right heart often leads to right-sided congestive heart failure, superior vena cava obstruction and pericardial effusion. Typically in our case the tumor was located in the right atrium and presented as pericarditis. Presenting symptoms are not specific and include dyspnea, chest pain, cough, hemoptysis, syncope, and cardiac arrhythmias. The atypical clinical presentation, the rareness and the rapidly evolving nature of this malignancy, are responsible, at least in part, for the late diagnosis in almost all reported cases.

Imagine techniques have an important role in early diagnosis. TTE and especially, transesophageal echocardiography (TEE), can precisely locate the tumor, define its extent, and may accurately predict tumor type [6,7]. Some patients of course, as in the present case, have negative initial echocardiographic findings either because the tumor is too small or its location on the posterior surface of the myocardium prevents accurate assessment [6]. However, since recurrent pericardial effusion is a common clinical feature of cardiac angiosarcoma and pericardial constriction can occur as in a recently reported case [8], echocardiography is of great importance in differential diagnosis of an effusing-constrictive pericarditis.

CT and MRI have excellent diagnostic advantages with regard to tumor delineation and spread [3]. More specifically, MRI currently appears to be the imaging modality of choice in the assessment of a patient with known cardiac mass [9]. Although CT was thought to be less useful in cardiac tumors in the past [10], the current generation of 16 and 64-slice multidetector CT does allow gating to the cardiac cycle, being a valuable tool.

The main treatment strategy in cardiac angiosarcoma is surgical resection with or without chemotherapy and radiation. However, regardless of treatment, prognosis is poor with survival ranging from 6 to 12 months. Novel approaches, such as the use of interleukin-2, have been reported to be effective. In a case of cardiac angiosarcoma treated with a combination of chemotherapy and immunotherapy, survival of 30 months after surgery has been reported [11].

## Conclusion

Given the rarity of this fatal condition and the lack of a specific clinical presentation, the diagnosis of cardiac angiosarcoma requires a high degree of suspicion. Early diagnosis may facilitate the management of these patients.

## Competing interests

The author(s) declare that they have no competing interests.

## Authors' contributions

MK, JF and CR were involved with the patient's management. CC was the patient's surgeon and helped write the manuscript. DE provided the CT scan illustrations and reviewed the manuscript. DR carried out histopathological analyses. MK and CR wrote and prepared the manuscript. All authors have read and approved the final manuscript.
